# Epidemiological monitoring survey to assess the impact of mass drug administration with triple-drug regimen in lymphatic filariasis elimination programme in an endemic district in Southern India

**DOI:** 10.1371/journal.pntd.0013368

**Published:** 2025-08-01

**Authors:** Kaliannagounder Krishnamoorthy, Raja Jeyapal Dinesh, Rajendran Dhanalakshmi, Priskilla Johnson Jency, Palappurath Maliyakkal Azad, Sugeerappa Laxmanappa Hoti, Manju Rahi, Ashwani Kumar

**Affiliations:** ICMR-Vector Control Research Centre, Puducherry, India; Cyprus International University: Uluslararasi Kibris Universitesi, CYPRUS

## Abstract

**Background:**

Mass Drug Administration (MDA) with triple-drug regimen (Ivermectin, Diethylcarbamazine, and Albendazole- IDA), recommended by the World Health Organization (WHO) was introduced in India in 2018, as an alternate strategy to two drug regimens (Diethylcarbamazine, and Albendazole- DA), for accelerating lymphatic filariasis (LF) elimination. By December 2023, IDA-MDA has been implemented in 63 LF endemic districts in India. The currently followed monitoring and evaluation (M&E) guidelines for DA may not be suitable to this new strategy. The WHO is developing a new M&E guideline for IDA-based MDA which recommends surveys referred to as Epidemiological Monitoring Survey (EMS) before the IDA Impact Survey (IIS), similar to pre-transmission assessment survey (preTAS) and transmission assessment survey (TAS) for DA-based MDA. The National Centre for Vector Borne Disease Control (NCVBDC) recently revised its M&E guidelines and downsized the evaluation unit to the health block from district level. In the present study, an EMS was conducted in two health blocks of Bidar district in Karnataka, India, in July 2023, following two effective rounds of IDA-MDA to assess its eligibility for an IIS.

**Methods:**

Two sentinel and one random site in each block were selected for the EMS, as per NCVBDC guidelines. A minimum sample of 300 individuals aged ≥20 years was tested for circulating filarial antigen (CFA) using filariasis test strips (FTS) in each site. Night blood smears were collected from CFA-positive individuals and examined for microfilaria (Mf). A Mf prevalence threshold of <1% in each site was used as a decision rule on whether to stop MDA and proceed with IIS or continue with MDA in the health block. The data were expressed as proportions with 95% confidence intervals, and a p-value <0·05 was considered statistically significant.

**Results:**

The number of individuals tested for CFA ranged from 303 to 314 in each of the six sites selected for EMS. The prevalence of CFA was above the threshold of 2% (3·6% to 31·1%) in all sites in both health blocks. Overall, 1846 individuals were screened, of which 343 (18·6%) were positive for CFA. Of the 320 individuals screened for microfilaremia, 71 were positive for Mf. All four sentinel sites in the two blocks had Mf prevalence above the threshold of 1%, ranging from 3·7 to 7·4%. The Mf count was 1–242 per 60µl of blood per positive person, and the geometric mean Mf density was 0·83 (±3·5) in the four sentinel sites. Both CFA (30·7%) and Mf (8·5%) prevalence was significantly higher in males. Nearly 42·0% of respondents self-reported not participating in both rounds of IDA-MDA, and it was significantly higher among males (52·4%). Also, the infection rates (CFA and Mf) were higher among those who did not participate in the two IDA-MDA rounds.

**Conclusions:**

Both the health blocks in Bidar district are not eligible for stopping MDA after two rounds of IDA-MDA, indicating the need for at least two additional rounds of MDA as per WHO guideline. The study suggests that the EMS strategy is operationally feasible which the other IDA-MDA districts can follow.

## Author summary

India has targeted the elimination of lymphatic filariasis (LF) by 2027, three years ahead of the global goal. To accelerate LF elimination, India introduced strategies of triple-drug regimen during mass drug administration (IDA-MDA) and Mission Mode MDA at the block level. IDA is more efficacious in clearing microfilaria (Mf) with fewer effective (coverage ≥65% among total population) MDA rounds (2–3) has been implemented in 63 districts as of December 2023 in India. A total of 138 districts have stopped MDA after clearing the first Transmission Assessment Surveys (TAS) or IDA Impact Surveys (IIS), and seven others have qualified for TAS/IIS following the detection of less than 1% Mf prevalence during pre-TAS/Epidemiological Monitoring Surveys (EMS). Here we report the findings from an EMS conducted in two health blocks to make decisions on stopping or continuing IDA-MDA. Both health blocks failed to qualify for the EMS, necessitating the continuation of MDA. The evidence generated will help the national programme to make decisions on continuing MDA in health blocks. Further, it will help to efficiently plan strategies that address the challenges faced during MDA to ensure effective coverage in areas at the highest risk of failing pre-TAS/ EMS.

## Introduction

Lymphatic filariasis (LF) is caused by filarial nematodes that are transmitted by mosquito vectors. The infection is mainly acquired in childhood and over time it leads to irreversible chronic manifestations of hydrocele and lymphoedema in some individuals if not treated [1]. The Global Programme to Eliminate Lymphatic Filariasis (GPELF) has targeted the elimination of LF as a public health problem, through Mass drug administration (MDA), by 2030. The GPELF has made significant progress, and by 2024, 21 of the 72 endemic countries achieved and validated LF elimination as a public health problem and are under post-validation surveillance [1,2]. MDA has been stopped in 12 more countries, and they are now under post-MDA surveillance while 39 other countries are still continuing MDA [1,2]. In 2017, the WHO recommended a triple-drug regimen (Ivermectin, Diethylcarbamazine, and Albendazole- IDA) for MDA as an alternate strategy to two drug regimen (Diethylcarbamazine, and Albendazole- DA), for accelerating LF elimination [3]. This strategy requires fewer rounds (2–3) of MDA and is more efficacious in clearing microfilaria (Mf) [4,5]. A total of 871 million people have been protected by GPELF, so far. However, as of 2024, 657 million people in 39 countries worldwide still require continuation of the preventive chemotherapy [1,2].

The Monitoring and Evaluation (M & E) of the programme to eliminate LF adopts the Transmission Assessment Survey (TAS) protocol as a decision-making tool for stopping MDA with a two-drug regimen and uses a critical cutoff value (CCV) of circulating filarial antigenemia (CFA) in 6–7-year-old children, determined by survey sample builder [6–8]. The size of the evaluation unit suggested is not more than two million populations. The ‘passing’ of a TAS means that the prevalence of LF in the evaluation unit has been reduced to a level at which transmission is probably no longer sustainable and recrudescence is unlikely to occur, even in the absence of MDA. A successful first TAS leads to the stopping of MDA. Repeat TAS (TAS-2 and TAS-3) with the same infection threshold is recommended at 2–3-year intervals for a post-MDA period of five years following the stopping of MDA [6–8]. This strategy has been widely used by the LF programme of endemic countries since 2011 and is considered a statistically rigorous and operationally feasible decision tool for evaluating the impact of MDA with two drug regimens [8,9].

**Fig 1:**
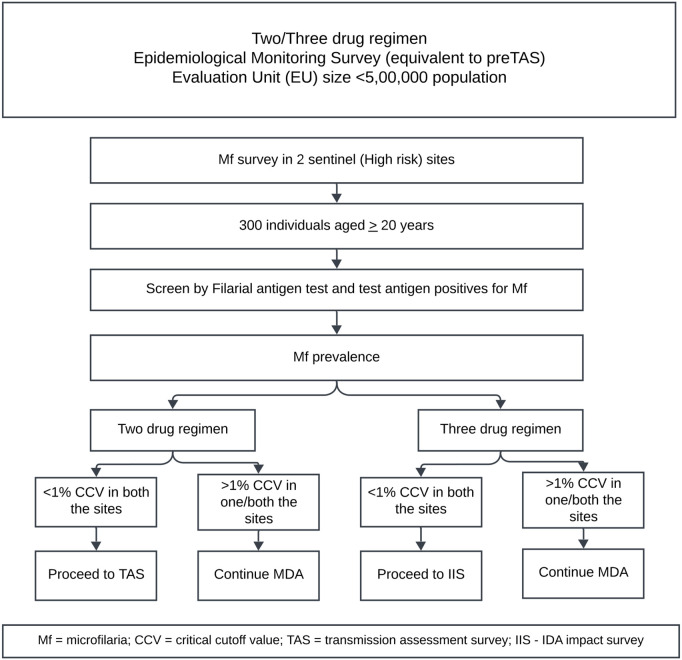
Flowchart showing revised WHO guideline for monitoring and evaluation of lymphatic filariasis elimination programme.

TAS is a decision-making tool that assesses filarial infection in terms of CFA among children aged 6–7 years. It is not suitable to evaluate IDA, which requires fewer annual rounds, unlike DA which requires 5–6 rounds [3,5,8]. Also, IDA is not very effective in reducing CFA but more efficacious in clearing microfilaria; hence, Mf can be a suitable indicator than CFA to assess the impact of IDA [4,10]. WHO has developed a provisional monitoring and evaluation guideline for IDA-based MDA and is used by 11 countries which introduced IDA. This includes the Epidemiological Monitoring Survey (EMS) (pre-TAS in case of DA-MDA) to verify whether infection is reduced to the level of Mf prevalence of <1% threshold in an evaluation unit (EU) and thereby can proceed to the resource-intensive IDA impact survey (IIS). The EMS is used in an EU in at least two purposively selected sites with a high-risk of transmission (based on either high parasite prevalence, vector abundance or clinical disease) with a population of not more than half a million and must have completed at least two rounds of effective coverage (≥ 65% of total population) with IDA. EMS suggests testing by filariasis test strip (FTS) followed by a night blood survey in all FTS positives. When each site shows <1% Mf prevalence among 300 individuals aged ≥ 20 years, the EU qualifies for an IIS. Even if one of the three sites documents Mf  ≥1%, the EU fails EMS (Fig 1). In case of failure, the WHO recommends continuing two more additional effective rounds of IDA-MDA before repeating an EMS in the EU. The IIS examines a sample of individuals aged ≥ 20 years in 30 random clusters for Mf prevalence [11]. If Mf prevalence is less than 1%, IDA-MDA can be discontinued and monitored further for four years [11].

In 2018, the National Centre for Vector Borne Diseases Control (NCVBDC) Delhi, India introduced IDA-based MDA to accelerate the LF elimination in the country, starting with five districts and gradually upscaling to 63 districts by 2023 [12]. DA-MDA is being continued in 107 endemic districts. In January 2023, India introduced Mission Mode annual MDA campaigns according to which the MDAs would be conducted in two phases every year in endemic districts synchronized with National Deworming Days (February 10^th^ and August 10^th^) as a part of an enhanced five-pronged strategy [12]. This intended to bring unprecedented focus and synergy to the programme and mass visibility to improve community compliance to MDA drugs [12]. The national programme also downsized the evaluation units recently from district to subdistrict (health block) level with a population not more than 0.5 million for all areas under MDA, irrespective of the regimen followed [12]. The present study provides the results of an EMS conducted in two health blocks (evaluation units) of Bidar district in Karnataka state, India, endemic for *Culex quinquefasciatus*-transmitted bancroftian filariasis, to determine if the blocks qualified for an IIS.

## Methods

**Ethics Statement:** The study was approved by the Institutional Human Ethics Committee (IHEC-0122/N/J, dated 21/7/2022) and the Health Ministry’s Screening Committee (HMSC, April 2023). Surveys were conducted jointly with the state and district officers/staff of NCVBDC. Written informed consent in the local language was obtained from all the study participants. For people who did not have formal education, written informed consent was obtained in the presence of a literate witness in the community, available at the time of the survey. All procedures followed the pertinent rules and principles of the Declaration of Helsinki. Individuals found positive by FTS were treated by the district health office as per the national guidelines (Supervised treatment with single dose of DA or IDA on day one followed by 12 days of 6mg/kg/day DEC) [12].

**Fig 2:**
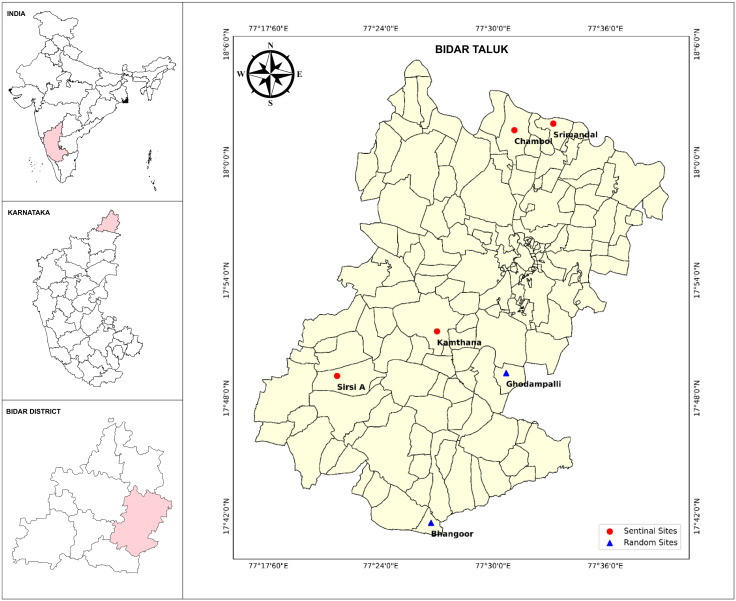
Maps showing the sentinel and random sites in two blocks in Bidar district of Karnataka, India. Base layer of map: https://onlinemaps.surveyofindia.gov.in. License information: https://onlinemaps.surveyofindia.gov.in/GeospatialGuidelines.aspx. ArcGIS Software: https://www.arcgis.com.

**Study area and design:** The Bidar district is located (17°35’ and 18°25’ North latitudes and 76°42’ and 77°39’ East longitudes) in the north-eastern part of Karnataka state, bordering the States of Maharashtra and Telangana (Fig 2). It has a population of about 1.7 million (Census 2011), spread over 5448 Km^2^ covering 8 taluks, 30 hoblies (a cluster of adjoining villages administered together for tax and land tenure purposes in the state of Karnataka), 635 villages and has an average literacy rate of 71%. The district is endemic for bancroftian filariasis and has undergone > 13 rounds of DA-MDA since 2004 [13]. Mane et al., (2018) reported 59.4% MDA coverage in 2016 and three other studies reported suboptimal coverage (52.2%, 60.4% and 63.4%) during the years 2007, 2011 and 2014 respectively in Bidar [13–16]. IDA-MDA was introduced in Bidar in 2021 to accelerate LF elimination. The district had completed two effective (reported coverage >80%) IDA-MDA rounds by 2023. The annual Mf survey conducted in one sentinel site and one random site reported <1% Mf prevalence and qualified for EMS as informed by the NCVBDC, Delhi. Bidar taluk (one of the 8 taluks) is administratively divided into 3 health blocks (Bidar A, Bidar B and Bidar C). A cross-sectional EMS was conducted in two of the health blocks viz., Bidar A and Bidar B.

**Selection of study sites**: In India, the evaluation unit of MDA is downsized to block level and is administratively managed by the district NCVBDC [12,17]. For assessment of impact of MDA, an evaluation unit will be constituted with a population not more than 5 lakhs. The block-level evaluation strategy of NCVBDC recommends the selection of two primary sampling units (PSU) as sentinel sites (purposive selection of high risk areas) and one additional random site for the EMS survey (Fig 1) [12,17]. The villages in rural areas and wards in urban areas are considered as PSUs. The sentinel sites are usually selected using data on the prevalence of either infection (previous records) or disease (morbidity data). As EMS is usually done by the respective State/district programme, the selection of the sentinel sites was done in consultation with the programme. The survey was technically planned by the research team and was conducted with active participation of the programme. Random sites were selected from a random number table after assigning numbers to the sites, excluding sentinel sites. In Bidar block A, two villages, Srimandal and Chambol, were selected as sentinel sites, recording 8 and 10 cases of lymphoedema (including hydrocele) respectively, whereas Ghodampalli was selected as the random site. In Bidar Block B, two villages, viz., Sirsi A and Kamthana 2, were selected as sentinel sites as these villages recorded the maximum number of lymphoedema cases of 16 and 14, respectively, and the Bhangoor village was selected as a random site. The location of the sites selected is shown in Fig 2. The population size of these selected villages is given in Table 1.

**Table 1:** Prevalence of CFA and Mf in sentinel and random sites in two health blocks of Bidar district.

Block/ EMS status	Site name	Site Type	Total population	Number of individual (≥20 years) tested (N)	Number Positive for CFA (a)	CFA% (95% CI) (a/N)	Smear taken (c)	Number Positive for Mf (b)	% Mf (95% CI) (b/N_a_)	Proportion Mf positive to CFA positive (b/c)
**Bidar** **Block A** **(EMS failed)**	**Srimandal**	**Sentinel**	1759	303	68	22·4 (17·7–27·1)	65	11	**3·7** **(1·5–5·8)**	16·9 (9·1–28·7)
	**Chambol**	**Sentinel**	3001	311	81	26·0 (21·2–30·9)	80	20	**6·5** **(3·7–9 ·2)**	25·0 (16·3–36·2)
	**Ghodampalli**	**Random**	2231	305	11	3·6 (1·5–5·7)	11	3	**1·0** **(0·0–2·1)**	27·3 (7·3–60·7)
**Bidar Block B** **(EMS failed)**	**Sirsi A**	**Sentinel**	3565	309	96	31·1 (25·9–36·2)	84	22	**7·4** **(4·4–10·4)**	26·2 (17·5–37·1)
	**Kamthana 2**	**Sentinel**	5450	304	65	21·4 (16·8–26·0)	59	15	**5·0** **(2·6–7·5)**	25·4 (15·4–38·7)
	**Bhangoor**	**Random**	1660	314	22	7·0 (4·2–9·8)	21	0	0·0 (0·0–0·0)	0·0 (0·0–0·0)
Total			17666	1846	343	18·6 (16·8–20·4)	320	71	3·9 (3·0–4·8)	22·2 (17·8–27·2)

In parenthesis: CFA- Circulating filarial antigen; Mf- Microfilaria; CI- Confidence interval, EMS- Epidemiological Monitoring Survey.

**Study population and sampling strategy:** From each site, at least 300 individuals aged ≥20 years were tested for CFA using filariasis test strips (FTS). Non-residents were excluded from the screening as per WHO guidelines (WHO defines a non-resident as someone who has lived in the area for < 1 year) [7]. Information regarding the surveys was conveyed to the panchayats and health sub centres in respective villages by the district health office a week prior to the survey. On the day of the survey, the health workers visited the houses in the respective area and informed the households of the survey and motivated them to participate in the survey. The screening was conducted during the daytime in camp mode. The camp site was organized in a centralized place (community centres, or health subcentres, or common public gathering points) in the village which was accessible to everyone. The two survey teams were stationed at different places on the three days so as to cover the entire village and achieve the sample size. The purpose of the study was explained to all eligible individuals voluntarily visiting the camp site. It was left to the discretion of the individual to decide whether to participate or not in the survey. The willing participants were enrolled after obtaining a written informed consent.

**Data collection and laboratory methods:** A new filarial rapid test kit, Q filariasis antigen test (QFAT), was evaluated along with FTS during the EMS in the four sentinel sites results of which is already published [18]. A finger-prick blood sample (75 μl for FTS and 20 μl for QFAT) was simultaneously drawn from the individual. Only the results of FTS tests are presented and discussed in this communication. To maintain the quality of tests (FTS and QFAT), house visits were avoided, which required carrying the diagnostic kits to all the selected houses. The test cards were labelled with a unique barcode. A semi-structured, pretested questionnaire that collected details on socio-demography and participation in previous IDA-MDA rounds was also administered to the participants. The data were also digitally collected using KoboToolbox - a survey platform for data collection, storage and visualisation with in-built data security features. One of the investigators was registered on this platform and created a customised template for data collection. This template was shared with the field workers, who used it for offline data collection using smartphones which were later synchronized with the online database. This data collection method was however, an adjunct to printed sheets to ensure that data-quality is maintained. The data collected through this platform were exported to an excel sheet which was accessible only to the investigator and cross-verified with hand-written forms prior to analysis. Each study participant was given a unique ID, and their personal information, such as name and address, were removed prior to data analysis to maintain anonymity and confidentiality.

The results of FTS for CFA were read exactly at 10 minutes. The scoring of the positive test was done based on the colour intensity of the test line, compared with that of a control line as suggested by the manufacturer. The scoring was as follows: strongly positive 3+ (test line stronger than the control line), moderately positive 2+ (test line as strong as the control line), and weakly positive 1+ (test line weaker than the control line). Invalid FTS tests were repeated once, and the repeat results were included for analysis. All FTS results were read by the card reader and cross verified by the team supervisor in the field to ensure quality.

The addresses of individuals positive for FTS were collected and their houses were visited after 21:00 hours the same day to collect night blood smears (60 µl) for Mf microscopy. Repeat house visits were made to sample the absentees of CFA-positive individuals. Three parallel lines, each with approximately 20 μl of blood, were prepared from each CFA-positive individual on a slide [6]. The slides were air-dried and stained using Giemsa following WHO guidelines [6]. The blood smear examination was done independently by two trained technical staff. All Mf positive slides and 10% of the negative slides were cross-checked by a senior technical staff. The survey in the two random sites was conducted in a similar camp mode by the district health team following similar methodology, and only the final results were shared with the research team.

**Statistical analysis:** Data entered in smartphones were cross-verified with the hard copies of the proforma filled while recruiting the participants. Data analysis was performed using Statistical Package for Social Sciences v21·0 (IBM SPSS Statistics Inc., Armonk, NY, USA). Each study participant was given a unique ID and their personal information such as name and address were removed prior to data analysis to maintain anonymity and confidentiality. The results were expressed as proportion with 95% confidence intervals. The CFA positive individuals who were not sampled for Mf even with repeat visits were excluded for calculating the Mf prevalence. Chi-square (χ^2^) test was used to find the association between the dependent (CFA, Mf) and independent variables (gender, participation in MDA). Non-parametric trend analysis was done to determine the association of CFA and Mf with age. A p-value <0·05 was considered statistically significant.

## Results

The total number of individuals who participated in the survey in each of the selected sites was between 303 and 314, and a minimum sample size of 300 was achieved in each site. A total of 1846 individuals aged ≥20 years participated in the survey and constituted 10·5% of the total Census population, in both the health blocks.

The number of CFA-positive individuals ranged from 11 to 96, and the corresponding prevalence fluctuated between 3·6% and 31·1% in different sites (Table 1). Thus, the CFA prevalence was above the 2% threshold in all sites. While one site was negative for Mf, all the remaining sites recorded Mf-positive cases and the number ranged from 3 to 22. Out of 343 CFA-positive individuals, night blood smears could be collected from 320 individuals. Despite repeated visits (two times), samples could not be taken from 23 (6·7%) CFA positive individuals as they either refused to cooperate or were unavailable at home at the time of the visit. The proportion of Mf positives among the CFA positives ranged between 16.9% and 27.3% in five sites and one random site recorded zero Mf positive. The Mf count ranged between 1 and 242 per 60 µl of capillary blood, and the geometric mean Mf density was 0·83 (±3·5). Both the blocks thus failed in EMS as the Mf prevalence was above 1% in one or more sites (Table 1).

The survey in the two random sites was conducted by the district health team following the same methodology, and only the summary results (shown in Table 1) were shared with the research team. Hence, further analysis was carried out only for the four sentinel sites which are presented below:

**Survey in sentinel sites:** A total of 1227 individuals were screened for CFA from the four sentinel sites. Females constituted 60·4% of individuals who participated in the survey. Almost all participants (99·6%) resided in the study areas for over three years. The study participants self-reported that 715 (58·3%) participated in the first round and 713 (58·1%) in the second round of IDA-MDA (Table 2). The proportion who participated did not statistically differ between the two rounds (χ^2^ = 0·0067; p = 0·934). It may be observed that only in Srimandal was the proportion who participated in MDA above 65% and the corresponding Mf levels was also lower compared to other sentinel sites (Table 2).

**Table 2:** Site-wise details on self-reported participation in MDA and Mf positivity rates in sentinel sites.

Bidar Block	Site Type	Site name	N	%Mf positive	% Participated in first round of IDA-MDA n (%)	% Participated in second round of IDA-MDA n (%)
**Block** **A**	**Sentinel**	**Srimandal**	303	3·7	215 (71.0)	217 (71.6)
	**Sentinel**	**Chambol**	311	6·5	138 (44.4)	138 (44.4)
**Block** **B**	**Sentinel**	**Sirsi A**	309	7·4	179 (57.9)	176 (57.0)
	**Sentinel**	**Kamthana 2**	304	5·0	183 (60.2)	182 (59.9)
**Total**			1227	5.6	715 (58.3)	713 (58.1)

**Non participation in IDA-MDA:** There were 509 (41·5%) individuals (~50% of either gender) who self-reported not participating in either of the IDA-MDA rounds. Of them, three were pregnant or lactating women. Their age characteristic was as follows: 20–29 years (25.1%), 30–39 years (19.1%), 40–49 years (14.7%), 50–59 years (12.8%), and 60 years & above (28.3%). Those who did not participate in two IDA-MDA rounds were significantly more among males compared to females. The proportion of CFA and Mf positivity were significantly (p < 0·001) higher among those who self-reported not participating when compared to those who had taken part in at least one round of IDA-MDA (Table 3).

**Table 3:** Association of CFA and Mf positivity with participation in IDA-MDA rounds.

Variable	Total n	Non participation in IDA-MDA a (% of n)	χ^2^	p value
**Male**	486	254 (52.3)	38.5	<0.001
**Female**	741	255 (34.4)		
**CFA positive**	509	164 (52.9)	22.3	<0.001
**CFA negative**	718	345 (37.6)		
**Mf positive**	500	43 (63.2)	14.0	<0.001
**Mf negative**	705	457 (40.2)		

**Reasons for non-participation in IDA-MDA:** The most common reason reported for non-participation (n = 469, multiple responses) was not being aware of MDA (72·6%), followed by refusal due to fear of drug side effects (9·9%), not being available at home at the time of drug distribution (9·7%), misbeliefs of not at risk of acquiring disease (3·6%), on medication for some other chronic illness (3·6%), and for other reasons such as bad taste of the drugs and religious beliefs (0·6%).

The proportion who did not participate in IDA-MDA was significantly (p < 0·001) higher in Chambol (55·6%) and Sirsi-A (42·1%) in Bidar blocks A and B, respectively and as was CFA and Mf prevalence (Tables 1 and 4).

**Table 4:** Gender-wise individuals who did not participate in the two IDA-MDA rounds in sentinel sites.

Bidar Block	Site Type	Site name	No. tested	#tested by gender		Non-participation in any of the two IDA-MDA rounds			p value
				#Male (n)	#Female (m)	Total (% out of N)	Male (% out of n)	Female (% out of m)	
**Block** **A**	**Sentinel**	**Srimandal**	303	104 (34·3)	199 (65·7)	85 (28.1)	44 (42·3)	41 (20·6)	<0·001
	**Sentinel**	**Chambol**	311	134 (43·1)	177 (56·9)	173 **(55.6)**	88 (65·7)	85 (48·0)	0·002
**Block** **B**	**Sentinel**	**Sirsi A**	309	131 (42·4)	178 (57·6)	130 **(42.1)**	71 (54·2)	59 (33·1)	<0·001
	**Sentinel**	**Kamthana 2**	304	117 (38·5)	187 (61·5)	121 (39.8)	51 (43·6)	70 (37·4)	0·29
**Total**			1227	486 (39·6)	741 (60·4)	509 (41.5)	254 (52·3)	255 (34·4)	<0·001

A higher CFA and Mf prevalence was observed in the older age groups. It may be noted that the CFA and Mf prevalence among individuals aged ≥60 years was as high as 33·0% and 9·9%, respectively (Table 5).

**Table 5:** Age-specific prevalence of CFA and Mf in the four sentinel sites in Bidar.

Age class (years)	FTS performed	#FTS positive (%)	Slides examined	#Mf positive (%)
20 – 29	308	49 (15·9)	305	8 (2·6)
30 – 39	247	65 (26·3)	242	11 (4·5)
40 – 49	200	53 (26·5)	199	13 (6·5)
50 – 59	172	44 (25·6)	165	7 (4·2)
60 & above	300	99 (33·0)	294	29 (9·9)
Total	1227	310 (25·3)	1205	68 (5·6)
Z value	–	3.4	–	3.6
p-value	–	<0·001	–	<0·001

**In parenthesis: CFA- Circulating filarial antigen; FTS- Filariasis test strip; Mf- Microfilaria.**

**Fig 3:**
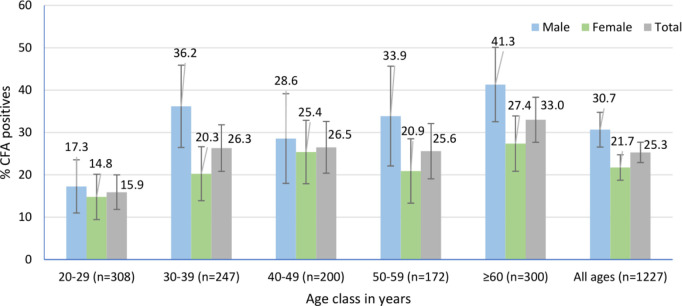
Age and gender distribution of CFA positives in the four sentinel sites in Bidar (n = 1227).

Fig 3 (S1 Table) presents the age and gender-specific prevalence of CFA in the four sentinel sites. Overall, the CFA prevalence was significantly (p < 0·001) higher in males (30·7%), compared to females (21·7%).

**Fig 4:**
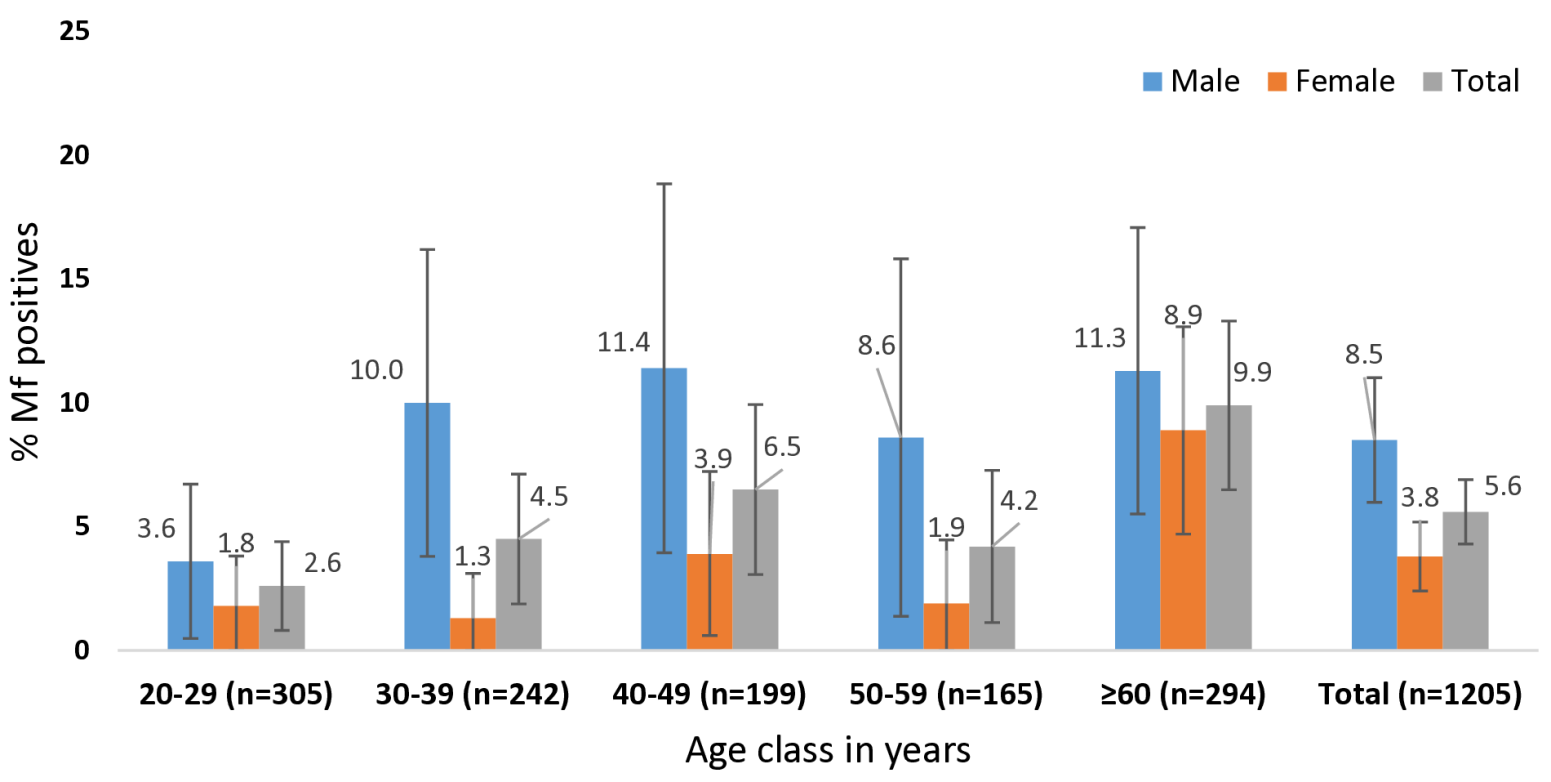
Age and gender distribution of Mf positives in the four sentinel sites in Bidar (n = 1205).

The age and gender distribution of microfilaremia in the four sentinel sites is shown in Fig 4 (S2 Table). Overall, the Mf prevalence was significantly (p < 0·001) higher among males (8·5%) compared to females (3·8%).

## Discussion

India is striving towards achieving the elimination of LF by 2027 [19]. To achieve this target, a revised MDA implementation strategy was launched in 2023 with Mission Mode MDA campaigns at block level twice a year synchronized with the National Deworming Days [12]. The remapping exercise was continued, and in 2023, three new districts were identified and brought under MDA [12]. MDA is stopped in 138 (40%) districts out of 345 endemic districts targeted for LF elimination and is continued in 174 districts (1701 blocks) as of December 2023 [12]. The IDA-based strategy is implemented in 63 districts, and 107 districts are under DA. Seven districts have cleared pre-TAS, and it is proposed in another 20 districts [12].

In 2022, the Bidar district in Karnataka state completed two effective rounds of IDA-MDA, and the annual Mf survey indicated that the district qualified for an epidemiological monitoring survey (EMS), as informed by NCVBDC. The EMS in Bidar was carried out following the block-level strategy in two evaluation units. The decision rule was that if any site recorded Mf prevalence ≥1%, the EMS would be considered failed and the block would continue with MDA [12,17]. The results showed that in Bidar block A, the Mf prevalence was above the critical threshold ≥1% in both sentinel and random sites whereas in Bidar block B, the Mf prevalence was above threshold in the two sentinel sites but it was zero prevalence in the random site. CFA prevalence, as high as 31%, was observed in one of the sentinel sites. Even in high infection prevalence settings, effective coverage should likely reduce Mf prevalence within a year, as observed during the IDA vs DA safety trial in Karnataka, India [4]. A study analyzing existing programmatic data from 13 countries to understand factors influencing pre-TAS results found that a higher prevalence of LF infection and 6 or more rounds of MDA were significantly associated with pre-TAS failure [20]. Although the reported coverage in the district was above 80%, the findings of the mandatory independent coverage evaluation surveys (CES) were not available for confirmation. One possible explanation for the persistence of infection in the present study is more likely due to suboptimal coverage (<65%). A study in Nagpur, India reported poor coverage in urban areas when compared to rural areas [21]. However, the two health blocks (EUs) studied were predominantly rural and a high coverage was expected. Seasonal population migration is yet another factor that may reduce the impact [22]. However, such patterns were not observed in the study blocks.

The WHO has provided a checklist to investigate the reasons for failure of transmission assessment surveys and recommended corrective actions to be taken to prevent and respond to failures [23]. Data on MDA coverage from independent evaluation surveys, data quality assessments, and supervisors’ coverage tools can be used to determine the reasons for suboptimal impact [23]. The present study clearly showed that a significantly higher proportion of males (52%) did not participate in IDA-MDA, and the infection rates in terms of CFA and Mf were also higher among them. This necessitates the development of novel strategies for their inclusion during the subsequent two additional MDA rounds for desired impact. It is also necessary to identify and address other potential barriers to participation, devise the most effective messages and channels for conveying health information, and devise effective drug administration strategies before undertaking the additional MDA rounds, with available tools to all GPELF partners [24].

Although the decision to qualify EMS is based on Mf rate, the guideline suggests testing for CFA followed by Mf among the CFA positives. This provides an opportunity to assess the CFA prevalence also in the EU, which was > 2% in all the sites, including the site that recorded Mf < 1%. This suggests that both IDA and DA regimens did not reduce CFA prevalence as reported elsewhere [4]. IDA is more efficacious in clearing Mf rapidly [4], it is expected that the proportion of Mf positives to CFA positives be reduced among those administered with IDA. None of the 21 CFA positives were Mf positive in Bhangoor, a site selected randomly irrespective of the risk status (no. of diseased LF cases). However, the results from the two purposively selected sites with high risk showed Mf prevalence of more than 1%. For programmatic decisions, it is necessary that Mf prevalence in each site is below 1%. Thus, although Mf was 0.0% in Bhangoor, the block failed EMS. It is necessary to continue with two more additional rounds of MDA before repeating an EMS. Accordingly, IDA-MDA was conducted in these blocks on the 10^th^ of August, 2023 [25,26]. The present study results are further suggestive of the high burden of LF infection persisting in both health blocks.

In the present study, the overall CFA prevalence was 18·6% after two rounds of IDA-MDA. A suboptimal impact is expected if the coverage is lower than the recommended 65%. During the annual Mf surveys conducted in 57 districts in 2022 in India, only 18 (31·5%) districts qualified for pre-TAS, according to the unpublished Joint Monitoring Mission (2022) report. Many districts had failed in the annual Mf surveys, pre-TAS and TAS, despite conducting annual MDA for several years. Among the five states that conducted TAS evaluations, 64 districts reported failure of TAS-1, 10 districts of TAS-2, and 2 districts of TAS-3 as per the report.

The WHO independent monitoring coverage assessment of the first round of IDA-MDA in 12 districts in India ranged from 25% to 84% [27]. Poor coverage is more likely to result in suboptimal impact. A modeling study has shown that the proportion of people who do not participate in MDA programmes for LF can strongly influence the achievement of elimination targets and that the impact is greater in high transmission areas [28]. They have shown that in *Culex-*transmission settings with a low (5%) baseline Mf prevalence and on either DA or IDA treatment, elimination can be reached only if treatment coverage among eligibles is 80% or higher. It is also documented that the rate of clearance of infection (CFA or Mf) is much slower among those who do not participate in the MDA rounds [29]. Sub optimal impact was also observed in some of the IDA districts during EMS after two rounds of IDA-MDA, as informed by NCVBDC. This is evident in the present study as well, with 41·5% of individuals self-reported not participating in the previous two IDA-MDA rounds. The filarial antigenemia and microfilaremia rates were also higher among those who did not participate in the IDA-MDA rounds. These untreated individuals may remain potential reservoirs, contributing to the sustained LF transmission in the community. Further, the majority (72·6%) who did not participate were unaware of MDA, and nearly 10% did not participate due to fear of the side effects. A study in Nagpur, India, reported that only 5% were unaware of MDA, whereas 13% did not participate due to fear of side effects [21]. This warrants intensive Information Education Communication (IEC) and social and behavioural change communication (SBCC) activities, before the MDA rounds to enhance community participation. Almost all (99%) of the participants reported residing in Bidar for more than 3 years and therefore had equal opportunity to benefit from MDA.

The preponderance of males for LF infection was evident from this study, corroborating the findings of earlier studies from India, Tanzania, and American Samoa [30–33]. Further, a higher CFA and Mf prevalence was observed in the older age groups, similar to the results from other studies [30–32]. It may be noted that nearly 28% of individuals aged 60 years and above did not participate in both of the IDA-MDA rounds and remained a potential reservoir, as reported by other studies [28,34]. Such high-risk groups (non-participants in MDA and elderly) can be identified and targeted, as advocated by Lau et al., 2020, and others [28,34,35].

While pre-TAS requires sampling at least 500 individuals aged ≥5 years [7], an EMS recommends sampling 300 adults aged ≥ 20 years. This change in age class has been proposed by WHO as adults are known to have a higher prevalence of Mf than children. This would improve the sensitivity of surveys to detect and respond to ongoing transmission. The introduction of CFA testing followed by Mf testing (among CFA positives) also makes the survey simpler and easier, as it avoids taking night blood samples from everyone. The reduction in sample size, change in target age class and the testing strategy make EMS operationally feasible. The results of an EMS conducted at a small number of sites, which costs far less than a TAS or IIS, are used to decide whether a full assessment (i.e., TAS/IIS) is warranted.

A recent modelling study using stochastic TRANSFIL model suggested reducing the Mf threshold for making decisions on stopping MDA from <1% to <0.5% for adults ≥20 years, under different treatment coverages and baseline prevalences, is more cost-effective and increases the likelihood of local LF elimination [36]. Yet another study showed that a Mf prevalence threshold of 0·5%, corresponding to TAS-3, results in ≥95% predictive value even when the MDA coverage is relatively low [10]. These modelling studies have shown that if the Mf threshold is reduced from 1% to 0·5%, the EUs passing the EMS would be a challenge. Likewise, lowering the CFA thresholds from 2% to below 1%, although reduces long term costs, may lead to an increased number of MDA rounds to achieve such a low prevalence threshold [37].

**Strengths and limitations:** This is the first study to report the results of an epidemiological monitoring survey to evaluate IDA-MDA in India. The EMS methodology is discussed in detail which is important for the evaluation of LF elimination programme both globally and nationally. The study also provides evidence for the selection of high-risk age group ≥ 20 years (more conservative) for Mf and the use of antigen test (FTS) prior to Mf testing to be more feasible and time-saving than night blood surveys. As the EMS was conducted in research mode, the quality of the survey was maintained, and it was ensured that the minimum sample size was met in each of the study sites. The study to some extent explored the reasons for EMS failure in the two health blocks. However, there are a few limitations, such as the availability of details on socio-demography, environmental factors, migration and other details from participants in the study sites would have enabled us to better understand the prevailing LF situation. In-depth interviews with the individuals who did not participate in MDA and community drug administrators would have provided deeper insights. Further, as the surveys were conducted in camp mode due to time constraints, there is a risk of selection bias owing to the voluntary nature of participation (self-selected sample) of individuals. As baseline Mf prevalence data was not available, the change in Mf prevalence could not be compared in these areas. The self-reported coverage is prone to recall bias as it was conducted more than six months after MDA. Also the data on coverage evaluation surveys by independent agencies like medical colleges (if available) would have helped in better understanding of coverage scenarios during previous two IDA-MDA rounds. The CFA negatives were not tested for Mf, as it is presumed that the probability of an Mf-positive individual testing negative for CFA is negligible.

## Conclusions

The epidemiological monitoring survey in the selected two health blocks indicated that the prevalence of Mf infection remained above the threshold (Mf > 1%) despite several rounds of DA and additional two rounds of IDA-MDA. Neither of the two blocks qualified for proceeding for an IDA impact survey, necessitating additional two effective rounds of IDA-MDA before repeating an EMS. Strategies and interventions (like test-and-treat strategy and intensive IEC/SBCC activities) to ensure increased participation of people, especially targeting the high-risk males and older age groups, are necessary in response to the failed EMS. This warrants more operational research to address how to improve compliance among the never participated, how to bridge the gap in participation by the adult male population and the gap in community coverage and compliance before we proceed with further rounds of MDA.

## Supporting information

S1 Table Age & gender distribution of CFA positives in the four sentinel sites in Bidar- provides the data for Fig 3. (XLSX)

S2 Table Age & gender distribution of Mf positives in the four sentinel sites in Bidar- provides the data for Fig 4. (XLSX)
